# A Case Report of Celiac Disease and Small Bowel Adenocarcinoma: Two Diseases With Similar Symptoms

**DOI:** 10.7759/cureus.82086

**Published:** 2025-04-11

**Authors:** Ana Oliveira, Gisela Gonçalves, Bárbara Paracana, Laura Baptista, Tatiana Rodrigues

**Affiliations:** 1 Internal Medicine, Unidade Local de Saúde da Região de Aveiro, Aveiro, PRT

**Keywords:** abdominal pain, celiac disease, diarrhea, iron-deficiency anemia, small bowel adenocarcinoma

## Abstract

Celiac disease (CD) is an autoimmune disease triggered by gluten in the diet. The association between CD and increased risk of neoplasia is described mainly in patients diagnosed with CD and aged over 40 years or during the first years after CD diagnosis. Patients who remain symptomatic after 12 months of gluten-free diet should be screened to exclude neoplasms, especially lymphoma and gastrointestinal neoplasms. We report a case of small bowel adenocarcinoma (SBA) in a patient diagnosed with CD one year earlier.

## Introduction

Celiac disease (CD) is an autoimmune disease triggered by dietary gluten and is characterized by chronic enteropathy and multiorgan involvement [1]. CD can present with different clinical pictures, including chronic diarrhea, constipation, iron-deficiency anemia, arthralgia, and intestinal lymphoma. The diagnosis of CD consists of clinical, serological, and histopathological findings. Patients with refractory CD are those with a preexisting diagnosis of CD and who maintain symptoms despite a strict gluten-free diet for more than 12 months. If refractory CD is suspected, it is mandatory to perform upper endoscopy with biopsies to observe the intestinal mucosa for changes compatible with the disease or suggestive of neoplasia. This is important because approximately 50% of these patients develop lymphoma within five years of diagnosis [2].

One of the most recent national cohort studies in Sweden found an increased risk of CD patients developing malignancies, especially when they are diagnosed after the age of 40 years, and a higher prevalence of lymphoma, oropharyngeal and intestinal cancer [1,3]. In CD, there are several mechanisms that contribute to the pathogenesis of SBA, such as chronic inflammation, increased permeability to carcinogens, malabsorption of anticarcinogenic substances, and impaired immune response [4]. We report a case of small bowel carcinoma in a patient diagnosed with CD one year before.

## Case presentation

The patient was a 63-year-old female who was diagnosed with celiac disease in January 2021. At the time of diagnosis, she had daily diarrhea associated with weight loss and microcytic anemia. After starting a gluten-free diet, she reported a decreased frequency of stools and maintenance of body weight, but continued to have iron-deficiency anemia. In January 2022, she went to the emergency department due to abdominal pain in the lower left quadrant associated with nausea. She also reported worsening diarrhea patterns over the past few days, describing around 10 daily episodes of liquid stools, without blood or mucus. No fever or other symptoms were described.

**Figure 1:**
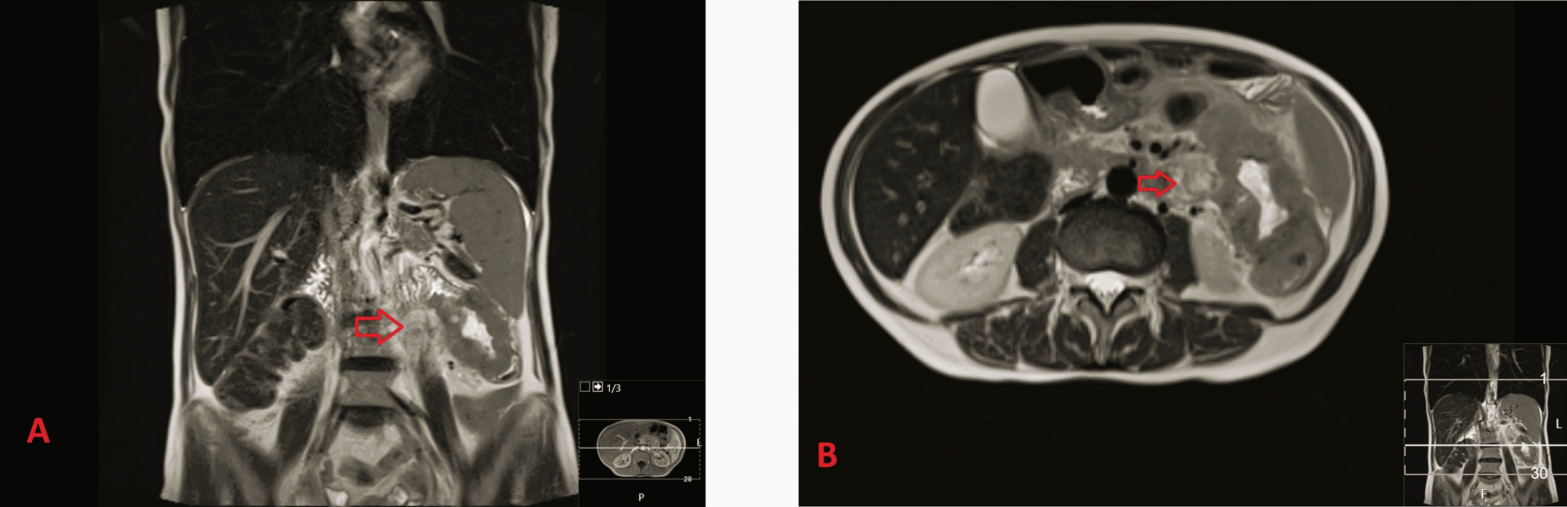
Bowell magnetic resonance. The images show neoplastic lesion located in the jejunum, without evidence of metastatic disease (A) and thickening of the proximal jejunum related to neoplasia (B). The arrows indicate the tumor lesion in the coronal axis (A) and in the axial axis (B).

On physical examination, she had pain on abdominal palpation, especially in the left hypochondrium, but without signs of peritoneal irritation. Laboratory studies demonstrated iron-deficiency anemia (Hb 7.1 g/dL, volume globular médio {VGM} 69.1 fL, iron 12 μg/dL, ferritin 23 ng/mL) and C-reactive protein (CRP) 6.08 mg/dL. Abdominal ultrasound identified a kidney-shaped mass in the small intestinal loop measuring 70 mm in diameter and infiltration of the surrounding fat. She was admitted to the internal medicine department for further investigation. Bowel MRI revealed a dilated proximal jejunal loop with parietal thickening, which could be classified as lymphoproliferative disease (Figures 1A, 1B).

**Figure 2:**
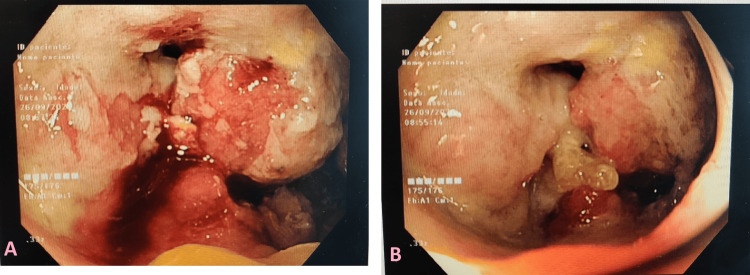
Enteroscopy showing small bowel findings. The images show an ulcerated exophytic lesion (A) and stenosis in the proximal jejunum (B).

**Figure 3:**
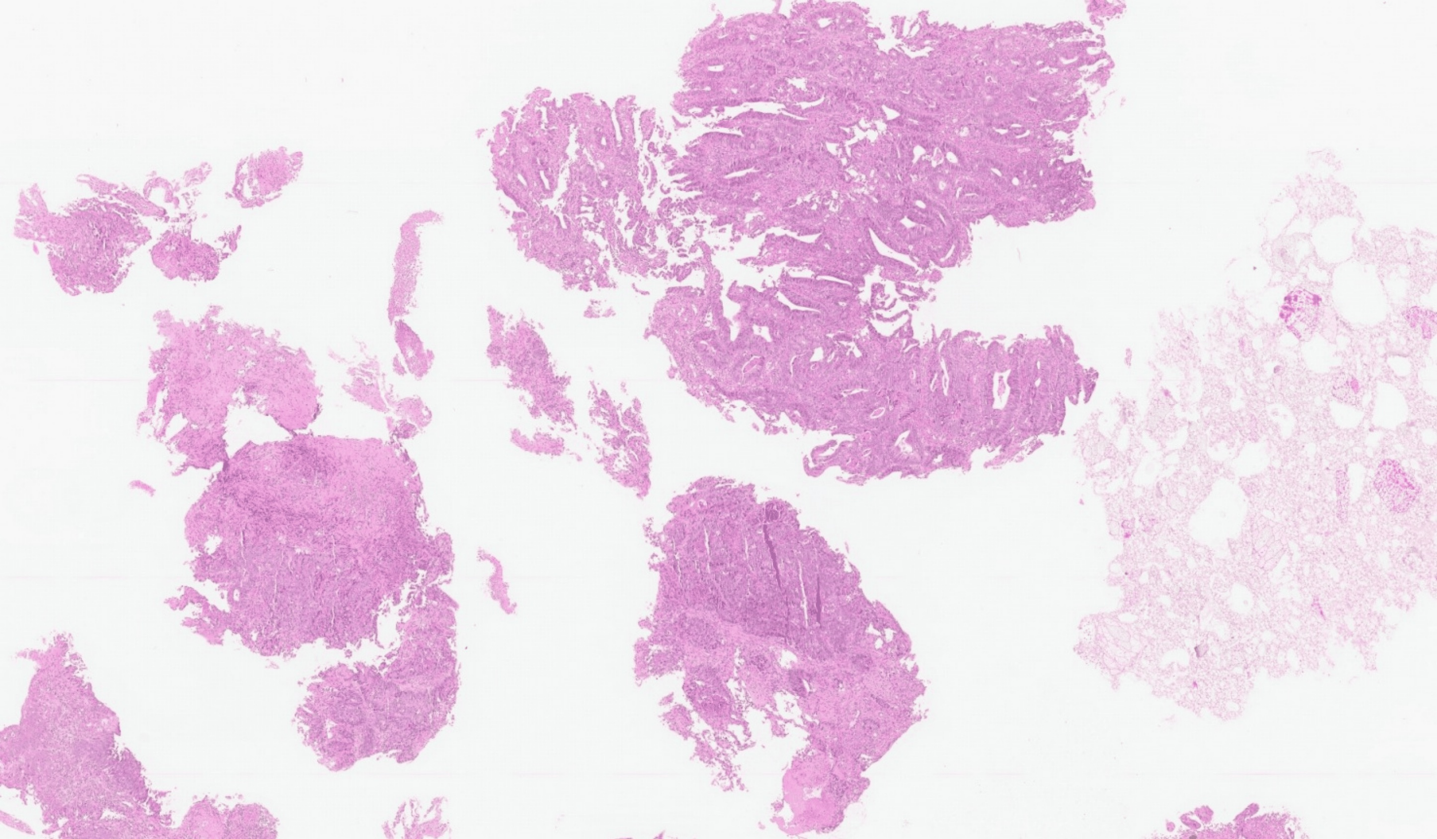
Histopathological findings of the jejunal biopsy. Moderately differentiated adenocarcinoma with infiltration of the intestinal mucosa (hematoxylin and eosin staining). This neoplasm presented predominantly tubular and solid architectural growth patterns. Cytology revealed pleomorphism, hyperchromasia, and anisokaryosis.

No distant lesions suggestive of metastasis were identified on MRI. The patient underwent an enteroscopy, which showed an exophytic, ulcerated, and stenosing lesion in the proximal jejunum (Figures 2A, 2B). Histopathological examination confirmed adenocarcinoma, and further workup revealed no evidence of metastasis (Figure 3). Therefore, the patient's case was discussed at a multidisciplinary group meeting to decide which therapy to pursue. After detailed analysis of the case and review of complementary diagnostic exams, the patient was proposed for neoadjuvant chemotherapy, because the tumor was not resectable by surgery. The patient did not want to start chemotherapy; therefore, she was referred to the palliative care team for supportive treatment. She died approximately three months after diagnosis.

## Discussion

In recent years, there has been an increase in studies investigating the relationship between malignancies and patients with CD. According to the most relevant studies, there appears to be a higher overall risk of cancer compared to the general population [1,2]. However, it is worth highlighting the limitations found in the studies, since they were carried out in reference centers and with observation of a small number of cases [3,5].

Small bowel neoplasms are rare, accounting for only 3% of all gastrointestinal tumors in the general population. However, it is estimated that approximately 40% of small bowel tumors are adenocarcinomas. Some authors associate SBA with small bowel diseases, including familial adenomatous polyposis, Lynch syndrome, Peutz-Jeghers syndrome, and immune-related bowel disorders (Crohn's disease and CD) [5]. SBA is usually diagnosed at an advanced stage, and it is associated with a poor prognosis, with an overall five-year survival rate of 26% [6].

A cohort study conducted in Sweden reports that patients with CD have a higher risk of developing neoplasms, especially those diagnosed with CD after the age of 40 years. This study also found a greater predisposition to the appearance of certain neoplasms, such as lymphoma and oropharyngeal and intestinal cancer [1].

In 1937, Fairley and Mackie were the first authors to describe six patients with intestinal lymphoma and CD. The relationship found between CD and intestinal lymphoma is based on gluten-induced enteropathy. In intestinal lymphoma, the tumor most often presents in the jejunum and is usually multifocal with ulcerative lesions. Currently, chronic inflammation is the most likely explanation for the association between lymphoma and CD [5]. Since lymphoma is a neoplasm most frequently associated with celiac disease, in our case, lymphoproliferative disease was the first diagnosis considered for the intestinal mass identified in abdominal ultrasound.

Compared with lymphomas, SBA was approximately 10 times less common in patients with CD [7]. It has been demonstrated that chronic inflammation in CD leads to the destruction of enterocytes, which leads to premalignant changes and, consequently, an increased risk of neoplasia. Some authors have even found small bowel adenoma as a precursor lesion, thus describing the adenoma-adenocarcinoma association [1]. Nowadays, it is largely agreed that low compliance within the gluten-free diet is the major factor leading to the development of SBA in the first year of the disease. However, SBA has also been described in CD patients who were on a strict gluten-free diet, thus highlighting the importance of other predisposing factors in the development of SBA [4,5]. However, SBA has also been described in CD patients who were on a strict gluten-free diet, thus highlighting the importance of other predisposing factors in the development of SBA. For example, the gut microbiome and genetic factors can increase inflammatory activity independently of the presence of gluten. This inflammatory response damages intestinal epithelial cells, which increases the risk of carcinomas [1,2,4].

Clinically, SBA may present with abdominal pain, gastrointestinal bleeding, vomiting, intestinal perforation, weight loss, and anemia. That said, we can see that the clinical presentation of CD and SBA is similar, so a high level of suspicion is necessary to make the differential diagnosis. In our patient, the abdominal pain and diarrhea could be explained by CD, which could delay further investigation. However, as the patient reported only slight improvement with the gluten-free diet, it also made us think about other etiologies for the clinical picture presented. According to the literature, SBA is often diagnosed simultaneously with CD, especially in patients over 40 years of age at the time of diagnosis. What makes this case challenging is the fact that SBA was identified approximately 12 months after the diagnosis of CD.

Regarding treatment, as it is a rare entity, all therapeutic recommendations are based on the treatment of sporadic cases. Therefore, if the disease is in its early stages, surgery is the first line of therapy. In advanced stages, surgery plus chemotherapy is the gold standard [1,8].

## Conclusions

In conclusion, this case highlights that SBA can occur in patients with CD and may mimic CD-related symptoms. When the patient is symptomatic, compliance with the gluten-free diet must be confirmed. If the diet is followed, specific antibodies for CD must be measured, and endoscopy should be repeated. This study is important to reevaluate the intestinal microscopy and identify malignant lesions at an early stage. In the case described, despite the gluten-restricted diet, the patient had persistent abdominal pain, diarrhea, and anemia, which led to a new etiological study, where SBA was identified. Given the known association between neoplasia and CD, it is important to maintain this diagnostic suspicion when symptoms do not respond to a gluten-free diet, especially in patients diagnosed in adulthood and during the first year of the disease.

Generally, the prognosis in SBA is poor; however, there appears to be a better prognosis if the tumor is diagnosed early. Therefore, we believe that in patients with refractory CD, gastrointestinal neoplasia should always be investigated to diagnose neoplasm as soon as possible. Currently, no guidelines recommend routine neoplasm screening in patients with CD.
